# Heated humidification did not improve compliance of positive airway pressure and subjective daytime sleepiness in obstructive sleep apnea syndrome: A meta-analysis

**DOI:** 10.1371/journal.pone.0207994

**Published:** 2018-12-05

**Authors:** Ding Zhu, Mindan Wu, Yuan Cao, Shihua Lin, Nanxia Xuan, Chen Zhu, Wen Li, Huahao Shen

**Affiliations:** Key Laboratory of Respiratory Disease of Zhejiang Province, Department of Respiratory and Critical Care Medicine, Second Affiliated Hospital of Zhejiang University School of Medicine, Hangzhou, Zhejiang, China; University of Rome Tor Vergata, ITALY

## Abstract

**Introduction:**

We performed a meta-analysis on whether heated humidification during positive airway pressure (PAP) could improve compliance and subjective daytime sleepiness in obstructive sleep apnea syndrome (OSAS) patients.

**Materials and methods:**

We searched PubMed, EMBASE, Medline, Cochrane Library, Clinical Trials, Web of Science and Scopus from inception to Oct 29, 2017. We made meta-analysis on the all available randomized controlled trials (RCTs) which assessed effects of heated humidification intervention on PAP compliance and subjective daytime sleepiness, by subgroups of automatic adjusting positive airway pressure/ continuous positive airway pressure (APAP/CPAP) usage and patients with/without upper airway symptoms prior to PAP therapy.

**Results:**

A total of nine RCTs were evaluated finally in this meta-analysis. When all the studies were pooled, heated humidification did not improve PAP usage time [weighted mean difference(WMD) = 13.28, 95% confidence interval(CI): -5.85 to 32.41, P = 0.17] or Epworth sleepiness scale (ESS) score (WMD = -0.63, 95% CI: -1.32 to 0.07, P = 0.08). In terms of PAP usage time, heated humidification failed to enhance compliance in both APAP (WMD = 22.34, 95%CI: -21.08 to 65.77, P = 0.31) or CPAP subgroup (WMD = 11.09, 95%CI: -10.21 to 32.40, P = 0.31) and it was also ineffective among patients with upper airway symptoms prior to PAP therapy (WMD = 22.74, 95% CI: -7.77 to 53.24, P = 0.14) or without (WMD = 13.22, 95%CI: -35.84 to 62.29, P = 0.60). In terms of ESS score, heated humidification did not reduce ESS scores in both APAP (WMD = -1.59, 95% CI: -3.81 to 0.64, P = 0.16) or CPAP subgroup (WMD = -0.39, 95% CI: -1.16 to 0.37, P = 0.32) and it was also helpless among patients with upper airway symptoms prior to PAP therapy (WMD = -1.17, 95% CI: -3.10 to 0.75, P = 0.23) or without (WMD = -0.30, 95%CI: -2.25 to 1.66, P = 0.76).

**Conclusion:**

Heated humidification during PAP therapy improves neither the compliance nor ESS scores in OSAS patients, no matter what types of PAP or whether the patients had upper airway symptoms prior to PAP therapy. But to the population with upper airway symptoms and the APAP users, the conclusions were limited because of small sample size and possible selection bias. More attentions should be paid to these potentially possible benefited subgroups.

## Introduction

Obstructive sleep apnea syndrome (OSAS) is a chronic breathing disorder characterized by repeated collapse of the upper airway during sleep, leading to nocturnal hypoxaemia, nocturnal respiratory arrest, recurrent micro awakenings and excessive daytime sleepiness. An apnea hypopnea index (AHI) of more than five, measured by overnight polysomnography, with associated daytime symptoms is necessary for the diagnosis of OSAS [1]. Uncontrolled OSAS is associated with a higher risk of developing stroke, erectile dysfunction, cognitive impairment, depression and cardiovascular diseases especially hypertension. These diseases will lead to a worse quality of life[2–6]. Moreover, OSAS and related symptoms also pose a threat to public safety such as traffic safety and occupational safety, resulting in a huge socioeconomic impact [7, 8].

Positive Airway Pressure (PAP), as effective and non-invasive modality, is the gold standard treatment, and it is well-accepted by OSAS patients. However, the success or failure of the therapy depends on patient’s compliance. Good compliance is defined as more than 4 hours of nightly use. Unfortunately, 46 to 83% of patients have been reported to be non-compliant to PAP, which is a bottleneck in the increasing success rate of PAP[9]. Many factors may cause the poor compliance, such as upper airway symptoms (blocked nose, dry nose/mouth/throat and so on), pressure intolerance, cold sensation of airflow and machine noise[10]. Based on these factors, researchers are focusing on making tentative interventions to raise compliance[9].

Heated humidification is one of such tentative interventions. Heat and moisture exchange is one of the most important functions of the respiratory system[11]. However, the application of PAP disrupts this function, because it causes high unidirectional nasal airflow, progressive drying of the upper airway mucosa, release of inflammatory factors, enhanced nasal mucosal blood flow and increased nasal resistance[12–14]. Interestingly, evidence suggests that heated humidification during PAP can reduce nasal resistance, lower the level of nasal lavage cytokines, attenuate inflammation and fibrosis of the nasal mucosa[15]. Hence, many scientists are looking forward to see that heated humidification can be used in PAP and produce some favorable effects to OSAS patients.

However, the conclusions about the effect of heated humidification on PAP compliance, subjective daytime sleepiness were inconsistent. Although at beginning, Massie et al reported that compared with CPAP alone, CPAP with heated humidification was associated with significant increase in compliance, greater treatment satisfaction and more refreshed feeling upon awakening[16], several researchers raised objections later on. They demonstrated that actually the increased hour of PAP usage result from heated humidification was not significant, which meant the compliance was still low with heated humidification [17–19].

Considering the controversial conclusions and the small sample size of each study, it is necessary to perform a systematic review and meta-analysis to further clarify the effects of heated humidification on PAP compliance and subjective daytime sleepiness in patients with OSAS. In our meta-analysis, we put emphasis on two outcomes from included studies, PAP usage hours and Epworth sleepiness scale (ESS). The data of PAP usage hours are obtained from PAP device recording the nightly duration of PAP therapy at effective pressure, which are used to assess the patients’ compliance. ESS are known as the most common self-administered questionnaire to assess subjective daytime sleepiness, so that it is widely used in screening OSAS and estimating curative effect after the therapy of PAP[20, 21].

## Materials and methods

This systematic review and meta-analysis was performed strictly in terms of the Preferred Reporting Items for Systematic Reviews and Meta-Analysis (PRISMA) guidelines[22]. And there was no protocol for this systematic review.

### Data sources and search strategy

Computerized and manual searches were carried out to identify all relevant researches. We searched the electronic databases including Pubmed, Embase, Medline, Cochrane Library, Clinical Trials, Web of Science and Scopus. The time frame ranged from the databases’ inceptions until Oct 29, 2017. The following search terms were used in the search for eligible studies: (“OSAS” or “Obstructive sleep apnea syndrome” or “OSA” or “Obstructive Sleep Apneas” or “Positive airway pressure”) and (“Heated humidification” or “Heated humidifier” or “Heated humidity” or “Heated breathing”). The reference lists attached to relevant articles were also reviewed strictly to identify eligible studies.

### Selection criteria

The studies were included if they satisfied the following criteria: (1) The population of studies were limited to adults with OSAS diagnosed by polysomnography according to the result of AHI ≥5 events/h. (2) OSAS patients in intervention groups received PAP with heated humidification; subjects allocated in the control groups received PAP without heated humidification. (3) All the studies which evaluated either or both of PAP compliance and ESS scores were included. (4) The study design had to be randomized controlled trial (RCT). (5) The treatment duration of the studies must be more than one week. (6) Only English language studies were included. Specially, one of the eligible articles was found by us owing to its abstract published in English, but the full text was written in Portuguese[23]. We tried to translate the article into our language by using online translator, and it turned out that we were able to understand the content well. Thus, we decided to bring into this article after a discussion of research team.

### Quality assessment

The recommended tool to evaluate the risk of potential biases of RCT was Cochrane Collaboration's tool[24]. Two authors (D.Z and M.D.W) used the Cochrane tool to distinguish studies and classify them as low, high, or unclear risk of bias, according to six domains: sequence generation, allocation concealment, blinding, incomplete data, selective reporting and other biases. Any discrepancy presented during the process of quality assessment was resolved by mutual agreement.

### Data extraction

Two independent authors (D.Z and M.D.W) identified potentially eligible articles by performing an initial screening of titles and abstracts. Then the authors made a further review by thoroughly reading the full texts to determine whether they should be included according to the above inclusion criteria. Two authors performed data extraction independently with a standardized data extraction worksheet and the third author confirmed its accuracy. The following data were extracted: first author's name, publication year, country, study design, type of PAP, diagnostic criteria of OSAS, basic data (age and gender), intervention treatment, control treatment, sample size, duration of treatment and outcome measures. If the outcomes were reported at different time points, we extracted data from the last time point. Any disagreement appeared between authors were resolved by discussion until reaching a consensus. Otherwise, a third author would be consulted.

### Statistical analysis

We considered the main outcome measures as continuous variables and then used weighted mean difference (WMD) and 95% confidence interval (CI) to express the size of the heated humidification effect on PAP usage time and ESS scores with a random effects model. Subgroup analyses were based on the choice of two types of PAP devices or the choice of two populations (with/without upper airway symptoms prior to PAP therapy). Heterogeneity across the studies was assessed using the I^2^ statistic. I^2^ values of 25%, 50% and 75% represent low, moderate and high heterogeneity, respectively. When heterogeneity was found, we conducted sensitivity analyses by removing each study from the analysis to assess the changes in the I^2^ values and determine which studies contributed most significantly to the heterogeneity. Publication bias was detected in studies using Egger’s test and Begger’s test. All analyses were performed with Review Manager (Version 5.3, The Cochrane Collaboration) and Stata (Version SE12.0, Stata Corporation, USA). A P-value of <0.05 was considered to be statistically significant.

## Results

### Identification of relevant studies

**Fig 1:**
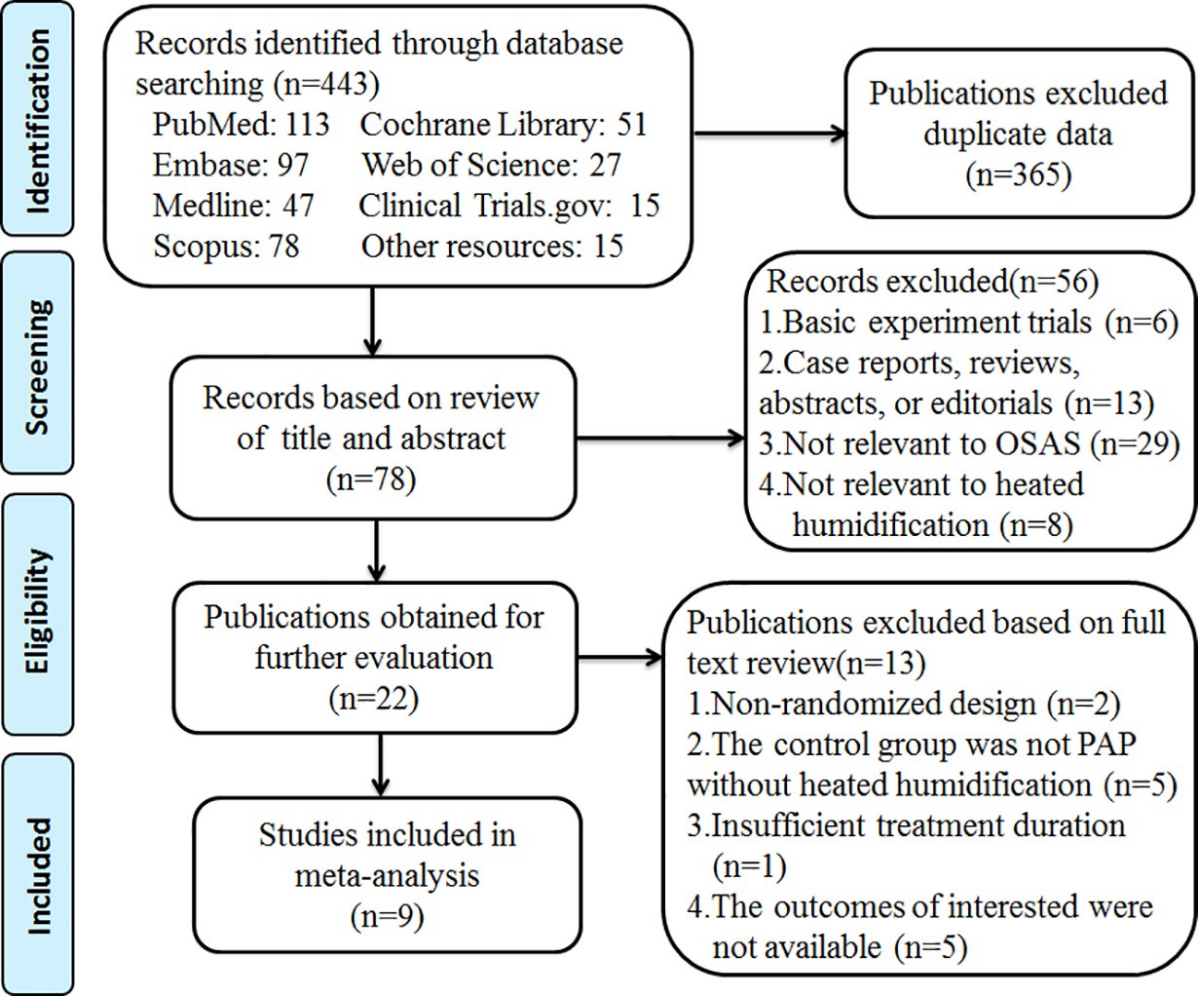
Flow diagram.

Our initial search of all databases retrieved 443 studies. After removal of duplicates, 78 records remained. After the review of titles and abstracts, a total of 22 studies were identified as relevant. On more detailed review by reading full texts, an additional 13 studies were excluded for the following reasons: not randomized design, inappropriate control group, not enough treatment duration and no outcomes of interested. Ultimately, only nine studies which satisfied the inclusion criteria were evaluated in this meta-analysis[16–19, 23, 25–28]. The detailed steps of the search are shown in (Fig 1).

### Characteristics of the studies

The details of study characteristics were summarized in Table 1. A total of nine studies met our inclusion criteria. Five studies were parallel RCTs[17–19, 23, 28], while the rest were crossover studies[16, 25–27]. The duration of intervention ranged from 3 weeks to 12 months. The mean age of participants from each study ranged from 44.1 to 59.9 years. The intervention groups received PAP with heated humidification, and the control groups received PAP without heated humidification. The nine studies included a higher proportion of men, ranging from 70% to 97%. Except that one study merely provided objective data of PAP compliance[26], the remaining eight studies provided both data of PAP compliance and ESS scores. The methodological quality of the nine studies was assessed according to the Cochrane tool in Table 2.

**Table 1:** The characteristics of included studies.

**Author**	**Study Design**	**Type (PAP/ Mask)**	**Average PAP pressure (cmH**_**2**_**O)**	**Symptomatic/ Asymptomatic Subjects**	**OSAS Severity (events/h)**	**Patients Gender (Male/Female)**	**Mean Age (years)**	**Number of Patients Analyzed**	**Treatment Duration**	**Intervention Treatment**	**Control Treatment**	**Outcome Measure**
**(year)**												
**Country**												
Massie[16]	RCT Crossover design	CPAP/ nasal mask	I1:9.5	both symptomatic and asymptomatic subjects	RDI	30/8	44.1±11	38	I1:3-week	I1:heated humidification I2:cold passover humidification	without heated humidification	1.PAP usage time (h/night) 2.ESS score
(1999)			I2:9.5		54.2±37.9				I2:3-week			
New Zealand			C:9.5						C:2-week			
Neill[25]	RCT Crossover design	CPAP/ nasal mask	10	both symptomatic and asymptomatic subjects	RDI	33/4	47.3±13	37	3-week	heated humidification	placebo humidification	1.PAP usage time (h/night) 2.ESS score
(2003)					50.1±25.7							
New Zealand												
Mador[17]	RCT	CPAP/ nasal mask	I:8.1	both symptomatic and asymptomatic subjects	AHI	I:47/2	I:59.9±11.5	I:39	12-month	heated humidification	without heated humidification	1.PAP usage time (h/night) 2.ESS score
(2005)			C:8.1		I:46.9±30.0	C:48/1	C:57.1±11.2	C:38				
USA					C:45.8±26.5							
Salgado[23]	RCT	APAP/ nasal mask	I:11.1	both symptomatic and asymptomatic subjects	AHI	I:13/4	I:57.4±9.2	I:17	30-day	heated humidification	without heated humidification	1.PAP usage time (h/night) 2.ESS score
(2008)			C:10.2		I:28.1±14.0	C:16/6	C:56.5±10.7	C:22				
Portugal					C:28.8±20.5							
Ryan[18]	RCT	CPAP/ nasal mask	I1:10	both symptomatic and asymptomatic subjects	AHI	I1: 40/2	I1:50±12	I1:39	4-week	I1:heated humidification I2:topical nasal steroid	without heated humidification	1.PAP usage time (h/night) 2.ESS score
					I1:36±20							
(2009)			I2:10		I2:35±21	I2: 40/2	I2: 48±12	I2:40				
Ireland			C:10		C:36±22	C: 36/3	C: 48±8	C:34				
Worsnop[19]	RCT	CPAP/ nasal mask	I:10.4	both symptomatic and asymptomatic subjects	AHI	I:21/4	I:55±11	I:25	12-week	heated humidification	without heated humidification	1.PAP usage time (h/night) 2.ESS score
(2010)			C:10.8		I:42±23	C:22/7	C:55±12	C:29				
Australia					C:50±20							
**Author**	**Study Design**	**Type (PAP/ Mask)**	**Average PAP pressure (cmH**_**2**_**O)**	**Symptomatic/ Asymptomatic Subjects**	**OSAS Severity (events/h)**	**Patients Gender (Male/Female)**	**Mean Age (years)**	**Number of Patients Analyzed**	**TreatmentDuration**	**Intervention Treatment**	**Control Treatment**	**Outcome Measure**
**(year)**												
**Country**												
Ruhle[26]	RCT Crossover design	CPAP/NA	NA	pure asymptomatic subjects	AHI	39/5	51.5±12.6	44	4-week	controlled heated humidification	without heated humidification	PAP usage time (h/night)
(2011)					43±28.1							
Germany												
Soudorn[27]	RCT Crossover design	CPAP/ nasal or full face mask	10.5	pure symptomatic subjects	AHI	14/6	48.9±12.7	20	4-week	heated humidification	without heated humidification	1.PAP usage time (h/night) 2.ESS score
(2016)					53.7±21.9							
Thailand												
Nilius(1)*[28]	RCT	APAP/NA	NA	pure symptomatic subjects	AHI	I:13/4	I:50.9±6.8	I:17	6-week	heated humidification	without heated humidification	1.PAP usage time(min/night) 2.ESS score
(2016)					I: 32.1±19.7	C:14/4	C:52.1±8.7	C:18				
Germany					C:27.6±19.0							
Nilius(2)^#^[28]	RCT	APAP/NA	NA	pure asymptomatic subjects	AHI	I:15/2	I:51.6±9.8	I:17	6-week	heated humidification	without heated humidification	1.PAP usage time(min/night) 2.ESS score
(2016)					I: 33.8±23.4	C:15/5	C:54.3±8.8	C:20				
Germany					C:25.7±16.4							

Data were shown as mean ± SD unless otherwise indicated. Definition of abbreviations: AHI = apnea hypopnea index; RDI = respiratory disturbance index; APAP = automatic adjusting positive airway pressure; CPAP = continuous positive airway pressure; PAP = positive airway pressure; OSAS = obstructive sleep apnea syndrome; RCT = randomized controlled trial; ESS = Epworth sleepiness scale; I = Intervention group; C = Control group;NA = not available.

Nilius(1)* and Nilius(2)^#^ were from the same study and were classified into two groups according to whether the population had upper airway symptoms prior to PAP therapy or not.

Pure symptomatic subjects: all subjects had upper airway symptoms prior to PAP therapy. Pure asymptomatic subjects: all subjects had no upper airway symptoms prior to PAP therapy.

**Table 2:** The quality assessment of included studies based on the Cochrane handbook.

Study	A	B	C	D	E	F	G	Total
Massie (1999)	?	?	**+**	?	**+**	**+**	**+**	4
Neill (2003)	?	?	**+**	**+**	**+**	**+**	**+**	4
Mador (2005)	**+**	?	**-**	?	**+**	**+**	**+**	4
Salgado (2008)	?	?	**-**	**-**	**+**	**+**	**+**	3
Ryan (2009)	?	?	**+**	**+**	**+**	**+**	**+**	4
Worsnop (2010)	?	?	**+**	**+**	**+**	**-**	**+**	3
Ruhle(2011)	?	?	?	?	**+**	**+**	**+**	3
Soudorn(2016)	?	?	**+**	?	**+**	**+**	**+**	4
Nilius(2016)	?	?	**+**	?	**+**	**+**	**+**	4

A = sequence generation; B = allocation concealment; C = blinding of participants and personnel; D = blinding of outcome assessment; E = incomplete outcome data; F = selective outcome reporting; G = other sources of bias; **+** = Yes;— = No; ? = unclear (‘Yes’ for a low risk of bias, ‘No’ for a high risk of bias; ‘Unclear’ otherwise)

### Meta-analysis

There were two types of PAP devices applied in the selected studies. Patients from two studies received automatic adjusting positive airway pressure (APAP) therapy and remaining eight studies received fixed continuous positive airway pressure (CPAP) therapy. Compared to fixed CPAP providing continuous fixed pressure throughout the night, APAP varies the pressure automatically depending on changes in airflow resistance during the entire sleep period. An updated meta-analysis of twenty-two trials showed APAP significantly improved patient compliance and reduced sleepiness as measured by the ESS compared with fixed CPAP[29]. Therefore, it seems to be reasonable to divide our selected studies into two subgroups: APAP group and CPAP group.

In addition, upper airway symptoms including dry nose, nasal congestion, runny nose, dry throat, sore throat, nose bleeds could affect the compliance of PAP[10]. So we paid more attention to the population with upper airway symptoms prior to PAP therapy. When using PAP, this special population might suffer an enhanced upper airway inflammation and more symptoms resulting in a higher risk in treatment intolerance because the application of PAP would act as a second-hit source of injury[14]. Fortunately, previous studies proved heated humidification can reduce the frequency of adverse upper airway symptoms[16–19, 25, 27, 28]. So we hypothesized the reduction of upper airway symptoms caused by heated humidification might lead to greater improvements in compliance and ESS scores, especially in the population with upper airway symptoms prior to PAP therapy. Hence, we tried to obtain the data of patients with and without upper airway symptoms prior to PAP therapy from all the studies.

### Subgroup meta-analysis

#### PAP usage minutes for compliance

Nine studies provided objective data from the PAP devices recording how long they were on at the effective pressure as an outcome to describe compliance. Average minutes of PAP use per night and SD were extracted and unified from all the studies.

**Fig 2:**
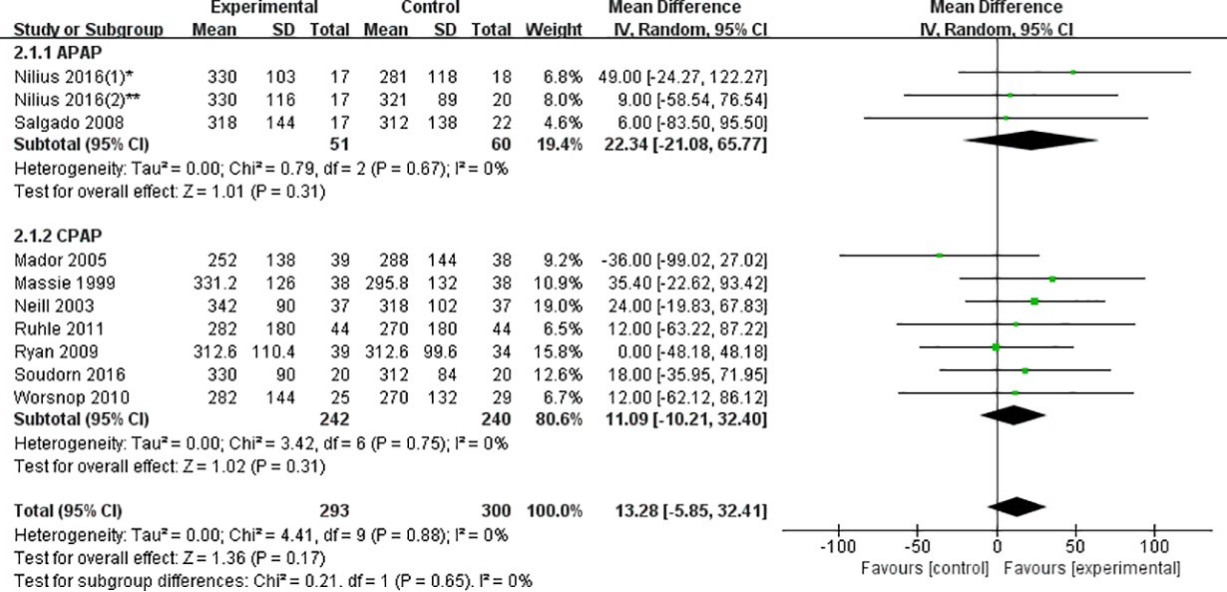
Forest plot of the association between heated humidification and PAP usage minutes according to the types of PAP.

By combining all included studies, the pooled effects of heated humidification on PAP usage minutes showed no significant differences (WMD = 13.28, 95%CI: -5.85 to 32.41, P = 0.17, I^2^ = 0%) (Fig 2).

**APAP group:** Two studies with three cohorts were included in the analysis. The pooled estimates of mean difference suggested no significant difference in minutes of APAP use per night between heated humidification group and control group (WMD = 22.34, 95%CI: -21.08 to 65.77, P = 0.31). And there were no significant heterogeneity (I^2^ = 0%, P = 0.67). (Fig 2).

**CPAP group:** We found no significant improvement in minutes of CPAP use per night between heated humidification group and control group (WMD = 11.09, 95%CI: -10.21 to 32.40, P = 0.31). The test for heterogeneity was not significant (I^2^ = 0%, P = 0.75) (Fig 2).

**Fig 3:**
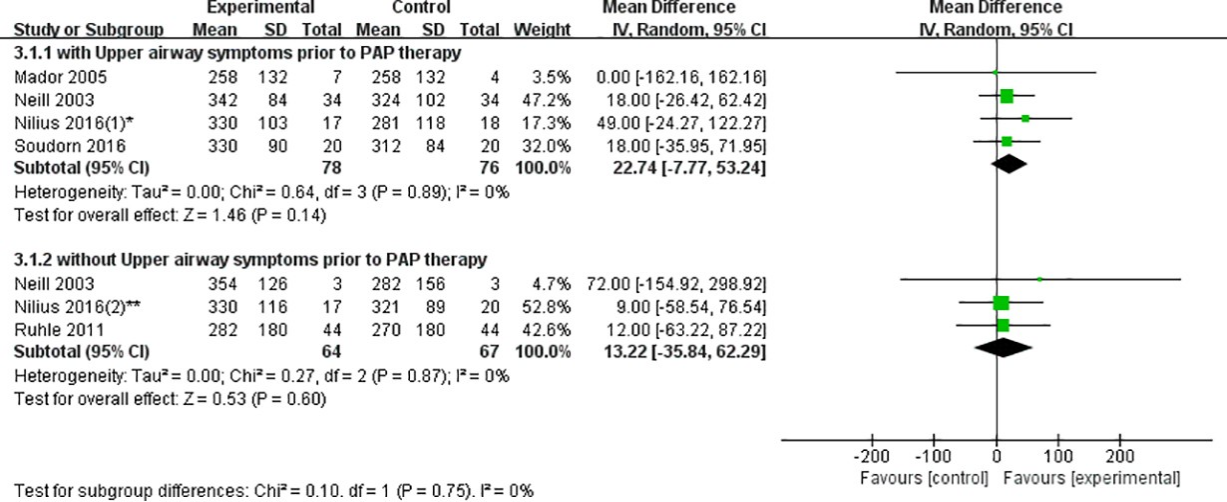
Forest plot of the association between heated humidification and PAP usage minutes according to patients with/without upper airway symptoms prior to PAP therapy.

**Patients with upper airway symptoms prior to PAP therapy:** Heated humidification group did not show any significant increase in the time of PAP use per night compared to control group in the pooled analysis (WMD = 22.74, 95% CI: -7.77 to 53.24, P = 0.14). The I^2^ test revealed no significant heterogeneity between the study outcomes (I^2^ = 0%, P = 0.89) (Fig 3).

**Patients without upper airway symptoms prior to PAP therapy:** The total WMD of the minutes of PAP usage in three studies with the patients who have no upper airway symptoms prior to PAP therapy is not significant, with a corresponding value (WMD = 13.22, 95%CI: -35.84 to 62.29, P = 0.60). There was also no significant heterogeneity (I^2^ = 0%, P = 0.87) (Fig 3).

#### ESS score

**Fig 4:**
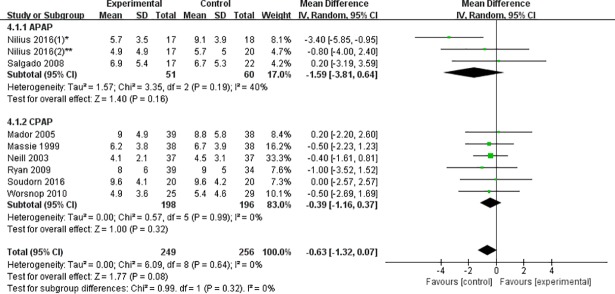
Forest plot of the association between heated humidification and ESS scores according to the types of PAP.

When all the studies were pooled, the total effects of heated humidification on ESS scores suggested no significant differences (WMD = -0.63, 95% CI: -1.32 to 0.07, P = 0.08, I^2^ = 0%) (Fig 4).

**APAP group:** Two studies including three cohorts provided available outcomes. There was no significant decrease in ESS score in heated humidification group compared to control group (WMD = -1.59, 95% CI: -3.81 to 0.64, P = 0.16). There was low heterogeneity (I^2^ = 40%, P = 0.19) (Fig 4).

**CPAP group:** In terms of ESS score, six eligible studies reported the sufficient data. Pooled analysis of the data revealed no statistically significant difference between heated humidification group and control group (WMD = -0.39, 95% CI: -1.16 to 0.37, P = 0.32). Significant heterogeneity was not found (I^2^ = 0%, P = 0.99) (Fig 4).

**Fig 5:**
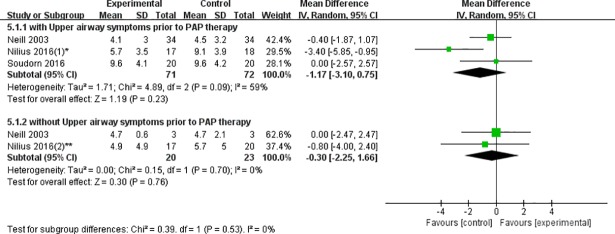
Forest plot of the association between heated humidification and ESS scores according to patients with/without upper airway symptoms prior to PAP therapy.

**Patients with upper airway symptoms prior to PAP therapy:** The values of ESS scores were extracted from three eligible studies for analysis. And there was no statistically significant decrease in ESS score between heated humidification group and control group (WMD = -1.17, 95% CI: -3.10 to 0.75, P = 0.23). But moderate heterogeneity was presented (I^2^ = 59%, P = 0.09) (Fig 5). Therefore, we tried to find out the source of heterogeneity by sensitivity analyses. Unfortunately, we failed to do it because there were only three studies which couldn’t meet the minimum study number demand of sensitivity analyses.

**Patients without upper airway symptoms prior to PAP therapy:** The total WMD of ESS scores with the patients who have no upper airway symptoms prior to PAP therapy failed to reach statistically significance (WMD = -0.30, 95%CI: -2.25 to 1.66, P = 0.76), and without statistical heterogeneity (I^2^ = 0%, P = 0.70) (Fig 5).

### Publication bias

**Fig 6:**
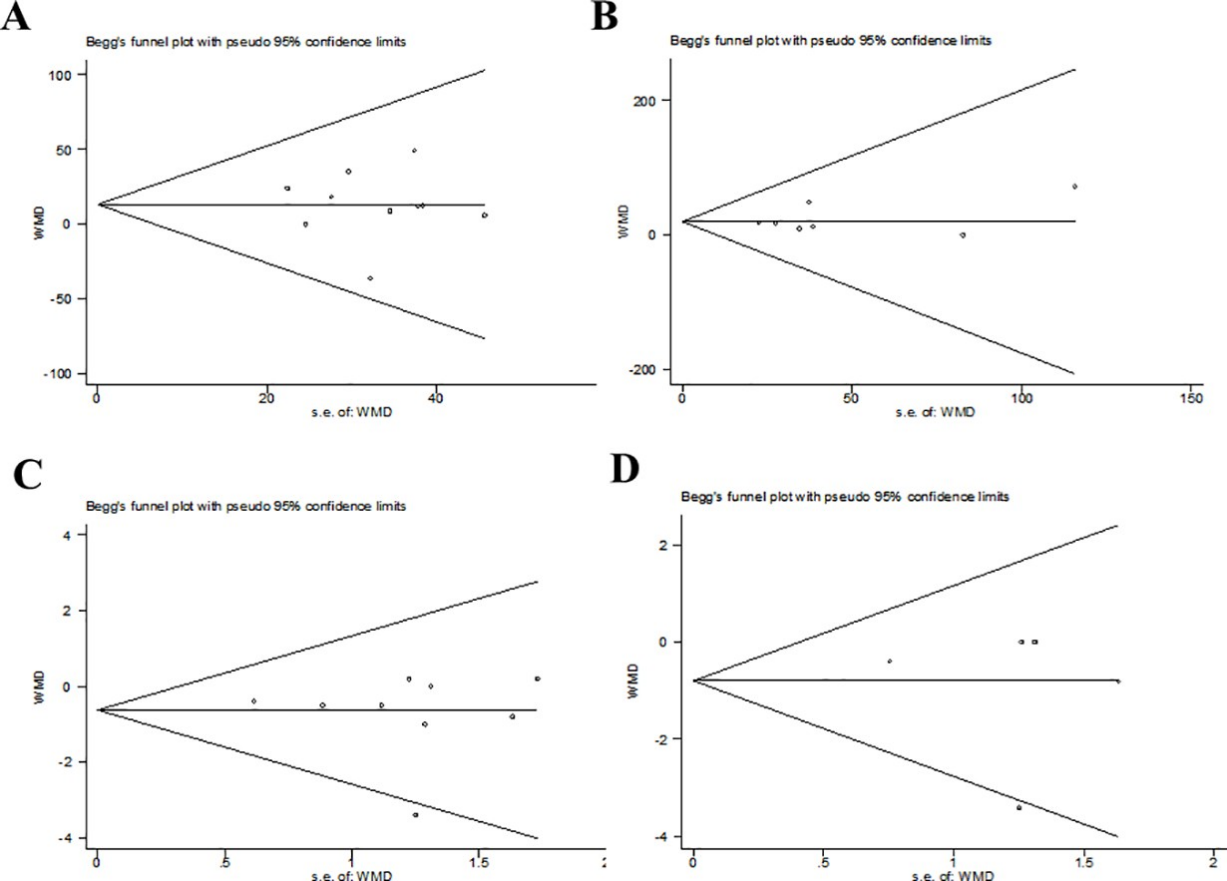
Assessment of publication bias by begg’ test using Stata SE12.0.

**Fig 7:**
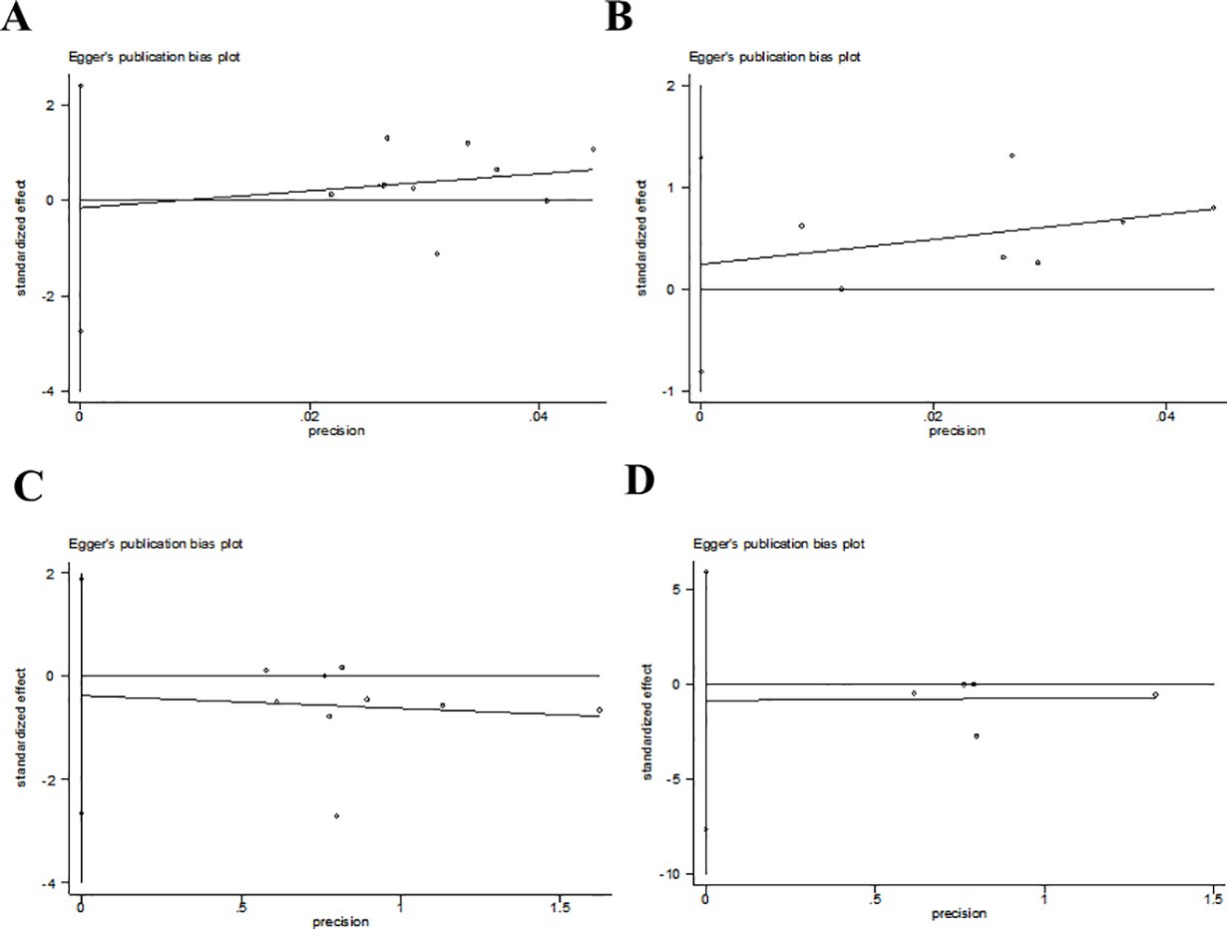
Assessment of publication bias by egger’ test using Stata SE12.0.

Publication bias was detected by Begg’s tests (Fig 6) and Egger’s tests (Fig 7). Overall, all of the included studies were deemed to have no risk of publication bias for all P values were more than 0.05 (Table 3).

**Table 3:** Publication bias form using Stata SE12.0.

Publication Bias	Included Studies	Begg’s Test	Egger’s Test
**PAP usage hours for compliance**			
PAP	9	0.858	0.892
symptoms prior to PAP	6	1.000	0.577
**ESS scores**			
PAP	8	0.917	0.706
symptoms prior to PAP	4	0.462	0.718

## Discussion

This is the first systematic review and meta-analysis to investigate the effects of heated humidification on compliance and subjective daytime sleepiness in OSAS patients using PAP. There were some major findings in this analysis. Heated humidification during PAP therapy improved neither the compliance nor ESS scores in OSAS patients, no matter what types of PAP or whether the patients had upper airway symptoms prior to PAP therapy.

Therefore, to the patients without upper airway symptoms prior to PAP therapy, we deemed that heated humidification was not necessary. But to the patients with upper airway symptoms prior to PAP therapy, we previously guessed heated humidification may improve their PAP compliance since plenty of studies had proved its effect on reducing the frequency of upper airway symptoms[16–19, 25, 27, 28]. Unfortunately, the result of subgroup analysis was beyond our expectation. Although the average difference in duration was around 23 min per night of PAP usage between heated humidification group and control group, data did not reach a statistical significance among patients with upper airway symptoms. We have several possible explanations for this unexpected result. First, most of current studies recruited unselected OSAS patients, without specifically focusing on preexisting upper airway symptoms. Only two studies (a total of 75 patients) took the population with upper airway symptoms as subjects investigated. Although we tried to obtain more original data by contacting the authors of each study to expand the sample size of patients with upper airway symptoms prior to PAP therapy, the sample size was still not large enough (154 patients). Similarly, the sample size of APAP group was also too small (111 patients). Such sample size might affect drawing a confirmed conclusion. Second, we supposed that the upper airway symptoms might not be primary determinant affecting compliance, and other factors such as the relief of OSAS symptoms and daily functioning seemed to be more critical to influence compliance[30–32]. Hence, the reduction of upper airway symptoms associated with heated humidification failed to translate into increased compliance.

The ultimate goal of improving PAP compliance is to improve the curative effect and reduce symptoms of sleepiness. The changes of ESS scores before and after therapy were considered as a reflex of curative effect in OSAS patients. Our results showed heated humidification during PAP did not reduce ESS scores, which meant heated humidification was not helpful to the curative effect.

### Possible mechanisms of heated humidification

The upper airway symptoms induced by PAP therapy may be associated with the following mechanisms. Firstly, animal experiments showed that nasal tissue compression caused by mechanical stimulus from PAP therapy triggered an early local inflammatory process, which resulted in neutrophil extravasation in the nasal mucosa[14]. Moreover, Constantinidis et al provided evidence to reveal that CPAP can destroy the equilibrium of the mucous membrane, giving rise to shedding and inflammation of the nasal mucosa[33]. Besides, PAP commonly exacerbates mouth leaks, causing high unidirectional airflow across the upper airway mucosa and drying it. The excessive drying of nasal mucosa has been proved to release vasoactive amines and leukotrienes which could increase mucosal blood flux leading to a rise in nasal resistance[12, 13].

Many upper airway symptoms caused by mechanisms mentioned above could be prevented by addition of heated humidification. Evidence indicated that heated humidification could decrease the level of nasal resistance and nasal lavage inflammatory mediators (IL-6, IL-12 and TNF-α), which can attenuate inflammatory cell infiltration and fibrosis of nasal mucosa[15]. Meanwhile, heated humidification could break the vicious circle produced by mouth leaks through reducing nasal mucosal blood flux and nasal resistance[12, 13]. Furthermore, Cruz et al demonstrated that it was cold and dry air instead of warm and moist air lead to epithelial cell damage and shedding on nasal mucosa[34]. Based on this finding, we have reasons to infer that the addition of heated humidification could avoid detachment and shedding of airway mucosa epithelial cell during PAP, thus relieving upper airway symptoms.

### Potential confounders and limitations

In our meta-analysis, we extracted two outcomes from included studies, PAP usage hours and ESS, which might cause some intrinsic confounders. First, ESS score, a subjective monitoring index, makes a rough estimate according to the changing trends of score to assess curative effect in OSAS patients. So that ESS score is not accurate enough. Second, some included studies were limited by inability to measure real PAP usage time because they didn’t describe whether the usage time under ineffective pressure was included or not. Third, the severity of OSAS wasn’t defined clearly in most of studies. However, patients with different severity of OSAS probably had different ESS scores because ESS scores were positively correlated with the AHI and negatively with the lowest SaO2[20]. For example, in Nilius et al study, there was a profound gap in AHI between heated humidification group and control group, which might generate interference to the results[28].

We also recognized that our analysis had other three limitations besides a relatively small sample size. First, researchers performed questionnaire investigation, maintained machines frequently in almost every study. Therefore, participants received fairly intensive follow-up, which might produce a potential encouragement or reminder to keep using PAP. Furthermore, it would be difficult to blind the patients which type of treatment they were getting, especially in long-term trials because patients had to perform the interventions (e.g. starting heating function) by themselves at home. In result, few studies met the demand of blind principles of clinical trials. Finally, some patients still had a certain chance appearing residual sleepiness even though they received standard PAP treatment[35], which would lead to elevated level of ESS scores and affect the results.

### Suggestions for further research

Although the use of heated humidification during PAP therapy was recommended by American Academy of Sleep Medicine[36], we make five suggestions for further research in this field.

First, the quantitative evaluation standard of heated humidification should be established in further studies, particularly the unified target values of temperature and humidity of the inspired air in the PAP tube system. And the time for increasing temperature and humidity to the target values should be taken into consideration in the same time. Because when the time is too short and temperature and humidity increase too quickly, it will condense the moisture into water in the mask which would negate the benefits of heated humidification on comfort and compliance.

Second, we expected that intelligent PAP devices which can not only perceive and monitor temperature and humidity of air in tube system, but also adjust the temperature and the humidity to the preset target value automatically should be introduced in future studies. At present, the tube system from the heater device to the mask is usually not heated or insulated in many studies. Hence, both heat and steam are easily to dissipate with the flow of air and therefore reduce the effectiveness of heated humidification, which may lead to negative results.

Third, future RCTs associated with heated humidification should focus on this special subgroup of OSAS patients. For example, the OSAS patients with at least one ENT (ear, nose, and throat) surgery or uvulopalatopharyngoplasty in the past is most likely to profit from heated humidification according to better compliance and improvement in daytime sleepiness and quality of life[28, 37].

Fourth, it is urgent to take some more objective assessment methods (such as multiple sleep latency test or maintenance of wakefulness test) other than the subjective indicator (ESS) to assess daytime sleepiness in future studies[38].

Lastly, we hope future studies could pay attention to side-effects of heated humidification. For example, heated humidification may potentially results in infections because of the pathogens colonization in the tube system theoretically. Moreover, it should be on alert that the flow of heated humid air through airway may evoke coughs and bronchoconstriction in the asthma patients combined with OSAS[39].

In summary, future research should be performed with longer follow-up period, lower risk of bias and report on adverse events to confirm the benefits and safety of heated humidification on PAP compliance and daytime sleepiness.

## Conclusion

Current studies indicated heated humidification during PAP therapy improves neither the compliance nor ESS scores in OSAS patients, no matter what types of PAP, whether or not the patients had upper airway symptoms prior to PAP therapy. But to the population with upper airway symptoms and the APAP users, the conclusions were limited because of small sample size and possible selection bias. More attentions should be paid to these potentially possible benefited subgroups in future.

## Supporting information

S1 File PRISMA checklist. (DOC)
